# Supplementary material for: The Impact of Lens Epithelium-Derived Growth Factor p75 Dimerization on Its Tethering Function
Source: Cells. 2024 Jan 25;13(3):227. doi: 10.3390/cells13030227 (PMC10854676; doi:10.3390/cells13030227)
Supplement: Supplementary file 1 [file cells-13-00227-s001.zip › cells-2731759-supplementary.pdf]

The Impact of Lens Epithelium-Derived Growth Factor p75 Dimerization on Its Tethering Function

Tine Brouns [1], Vanda Lux [2], Siska Van Belle [1], Frauke Christ [1], Václav Veverka [2,3] and Zeger Debyser [1,*]

[1] Laboratory for Molecular Virology and Gene Therapy, Department of Pharmaceutical and Pharmacological Sciences, KU Leuven, 3000 Leuven, Flanders, Belgium

[2] Institute of Organic Chemistry and Biochemistry of the Czech Academy of Sciences,16000 Prague, Czech Republic

[3] Department of Cell Biology, Faculty of Science, Charles University, 12800 Prague, Czech Republic

* To whom correspondence should be addressed. Tel: +32 16 375029; Email: zeger.debyser@kuleuven.be

SUPPORTING INFORMATION

Supporting Table S1. The different mutations were introduced via site directed mutagenesis using the primer combinations given in the table.

Name	Sequence
Fw1_LEDGFdNLS+Aths	5’ - CCT TCA GAG AGT GAC ATC ATT ACT GAA GAG G - 3’
Fw2_LEDGFdNLS+Aths	5’ - AAA ATG GTA AAA CAG CCC TGT CCT TCA GAG AG - 3’
Rv1_LEDGFdNLS+Aths	5’ - CCT TCT GGC AGC TTT TGG AGT AGT TAT GTC AAC T - 3’
Rv2_LEDGFdNLS+Aths	5’ - ACA GGG CTG TTT TAC CAT TTT CCT TCT GGC AGC TTT TGG - 3’
Fw1_LEDGF E451R-E452R	5’ - AAA ACC AAA GAT CAA GGG AAG AAA GGG CCA - 3’
Fw2_LEDGF E451R-E452R	5’ - CGG CAA CAC AGA AGG GCG AAT AAA ACC AAA GAT CAA - 3’
Rv1_LEDGF E451R-E452R	5’ - TTG TTC AGC AAG AGA TTT ATT CAG CAC TTG GGT - 3’
Rv2_LEDGF E451R-E452R	5’ - ATT CGC CCT TCT GTG TTG CCG TTG TTC AGC - 3’
Fw1_LEDGF$_{1-426}$	5' - GAT CCG GCT GCT AAC AAA GCC CGA - 3'
Fw2_LEDGF$_{1-426}$	5' - TAG AAT TCG AAG CTT GAT CCG GCT GCT AAC AAA - 3'
Rv1_LEDGF$_{1-426}$	5' - CAT GTT CTT AAA CTT GTT ATA CAA CAT TGT - 3'
Rv2_LEDGF$_{1-426}$	5' - AAG CTT CGA ATT CTA CAT GTT CTT AAA CTT - 3'
Fw1_d345_E451A	5' - AAT AAA ACC AAA GAT CAA GGG AAG AAA GGG CCA - 3'
Fw2_d345_E451A	5' - CAG CAT GCG GAA GCG AAT AAA ACC AAA GAT - 3'
Rv1_d345_E451A	5' - TCT TTG TTC AGC AAG AGA TTT ATT CAG CAC TTG GGT - 3'
Rv2_d345_E451A	5' - CGC TTC CGC ATG CTG TCT TTG TTC AGC - 3'
Fw1_d345_N454A_K455A	5' - GAT CAA GGG AAG AAA GGG CCA AAC ACT - 3'
Fw2_d345_N454A_K455A	5' - GAA GCG GCT GCA ACC AAA GAT CAA GGG AAG AAA - 3'
Rv1_d345_N454A_K455A	5' - TTT GGT TGC AGC CGC TTC CTC ATG CTG TCT - 3'
Rv2_d345_N454A_K455A	5' - CTC ATG CTG TCT TTG TTC AGC AAG AGA TTT ATT - 3'
Fw1_MBP325	5' - GTT TTA CAA CGT CGT GAC TGG GAA AAC CCT G - 3'
Fw2_MBP325	5' - TGA AAG CTT GGC ACT GGC CGT CGT TTT ACA ACG TCG TGA CTG GGA A - 3'
Rv2_d427_MBP325	5' - GAC GGC CAG TGC CAA GCT TTC AGA ACA TGT TCT TAA ACT TG- 3'
Rv2_d467_MBP325	5' – GAC GGC CAG TGC CAA GCT TTC ACT AGC TTT TTG TTT GGC C - 3'

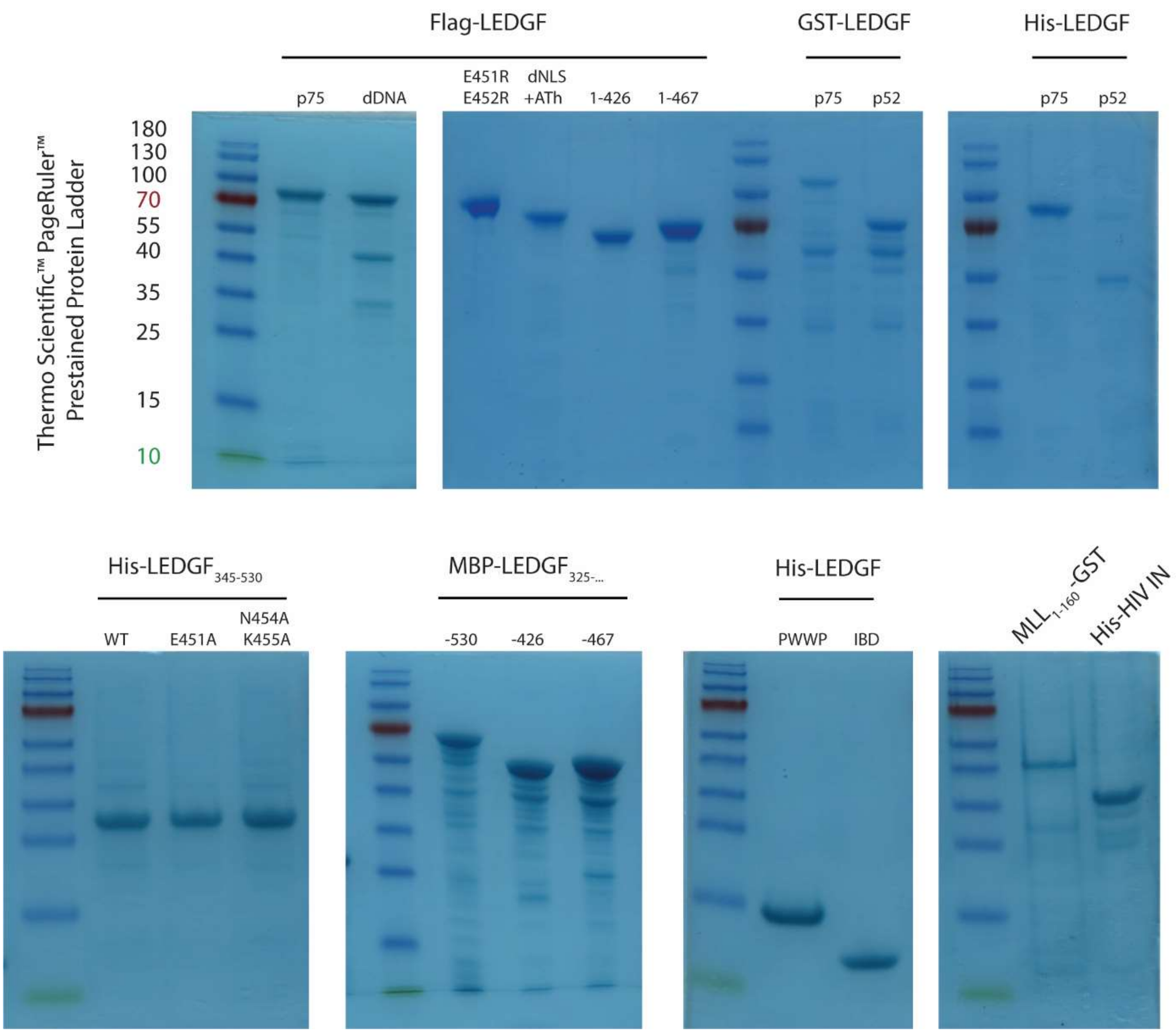

Supporting Figure S1. Coomassie of used proteins. To evaluate the purified proteins, 2 µg of each protein was loaded on an in-house 12% Tris-Glycine gel. Gels were run in Tris-Glycine buffer (TG) or MES buffer (M) in case of small proteins. The theoretical molecular weight is given between brackets below. Of note, LEDGF WT is known to run at a height of 75 kDa or 52 kDa instead of the theoretical 63 kDa and 41 kDa, for the p75 and p52 isoform respectively (hence the name). Gel 1 (top left) contains Flag-LEDGF/p75 WT (63 kDa) and Flag-LEDGF$_{dDNA}$ (63 kDa). Gel 2 (TG) contains Flag-LEDGF$_{E451R,E452R}$ (63 kDa), Flag-LEDGF$_{dNLS+ATh}$ (58 kDa), Flag-LEDGF$_{1-426}$ (52 kDa), Flag-LEDGF$_{1-467}$ (56 kDa), GST-LEDGF/p75 (86 kDa) and GST-LEDGF/p52 (65 kDa). Gel 3 (TG, top right) contains His-LEDGF/p75 (64 kDa) and His-LEDGF/p52 (41 kDa). Gel 4 (M, bottom left) contains His-LEDGF$_{345-530}$ WT and mutant proteins that al should run around 25 kDa. Gel 5 (TG) contains MBP-LEDGF$_{325-530}$ (69 kDa), MBP-LEDGF$_{325-426}$ (58 kDa) and MBP-LEDGF$_{325-467}$ (62 kDa). Gel 6 (M) contain His-PWWP (16 kDa) and His-IBD (13 kDa). Finally, gel 7 (M, bottom right) contains MLL$_{1-160}$-GST (43 kDa) and His-HIV integrase (IN; 34 kDa).

Supporting Table S2. DNA concentration in protein samples.

	µg/mL	
Flag-LEDGF/p75 WT	0.164	*±0.014*
Flag-LEDGF/p75 WT + MNase (1h)	0.055	*±0.002*
Micrococcal Nuclease	O.O.R.	
Flag-LEDGF(1-426)	0.042	*±0.004*
Flag-LEDGF(1-467)	0.087	*±0.016*
Flag-LEDGF(Ath mutant)	0.121	*±0.001*
Flag-LEDGF(dATh + NLS)	2.080	*±0.141*

N=2; MNase = Micorcoccal Nuclease; O.O.R. = out of range, value too low

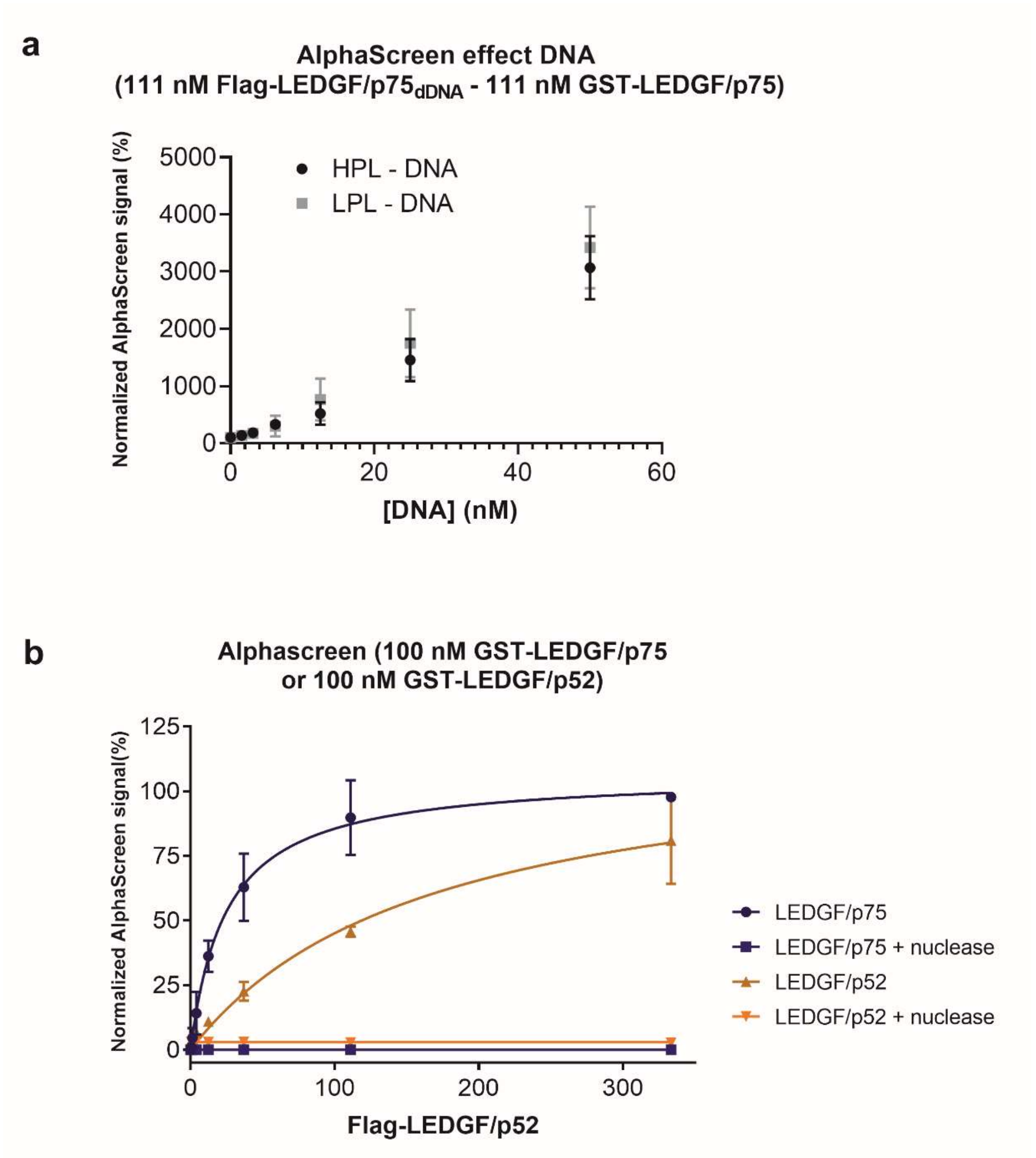

Supporting Figure S2. LEDGF dimerization is dependent on DNA. (a) Dimerization of Flag-LEDGF/p75dDNA to GST-LEDGF/p75 WT emerged possible in the addition of both high and low persistence length DNA (HPL and LPL, respectively). A higher DNA persistence length relates to lower DNA flexibility. One representative experiment is shown (n=2). Error bars represent standard deviation from duplicates. (b) Both the binding of LEDGF/p75 against LEDGF/p52 (purple curves), and the binding between LEDGF/p52 and LEDGF/p52 (orange curves) can be disrupted by adding nuclease into the AlphaScreen buffer, indicating that DNA is inducing the dimerization reaction for both LEDGF/p75 and LEDGF/p52. Error bars in all AlphaScreen experiments represent standard deviation calculated from at least two independent experiments.

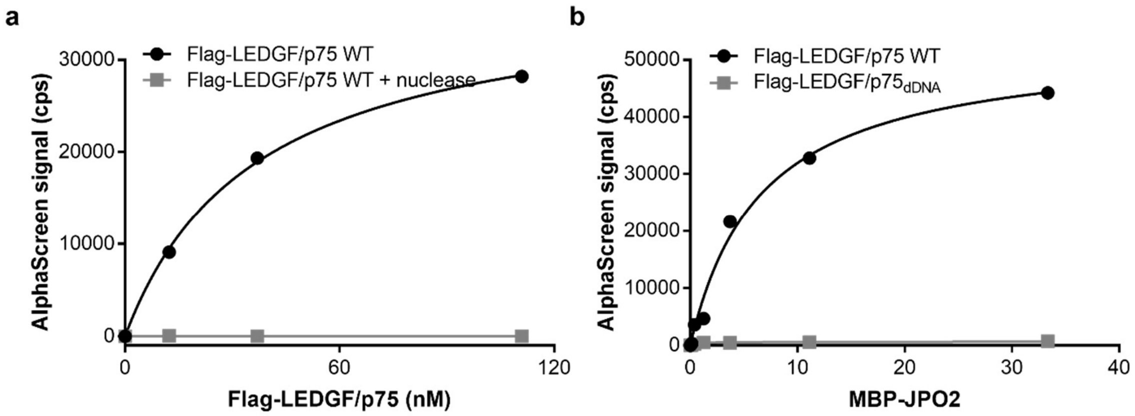

Supporting Figure S3. Binding of LEDGF/p75 to JPO2 is regulated by the presence of DNA. (a) Interaction of LEDGF/p75 WT with 33 nM MBP-tagged JPO2 in an AlphaScreen assay. Addition of 0.13 U/well micrococcal nuclease in the buffer (grey blocks) abolished the interaction between LEDGF/p75 WT and JPO2. (b) The interaction between JPO2 and 111 nM Flag-tagged LEDGF/p75 WT (black circles) or LEDGF/$p75_{dDNA}$ (grey blocks) is shown as well. These experiments were performed in singlet.

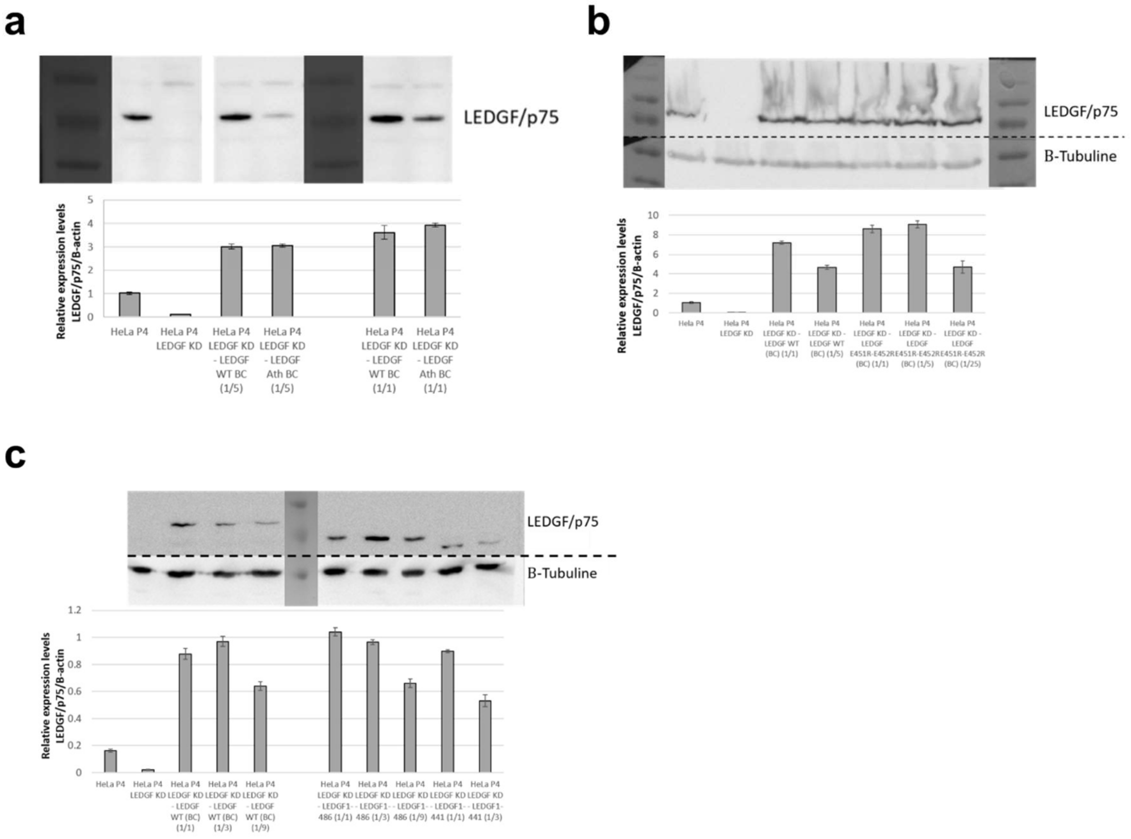

Supporting Figure S4. Expression levels of LEDGF/p75 in HeLa P4 cells by western blot and qPCR analysis. (a) Protein expression in HeLaP4, HeLaP4 LEDGF KD, HeLaP4 LEDGF KD – LEDGF WT/p75 (BC) and HeLaP4 LEDGF KD – LEDGF Ath (BC) are shown in western blot, while RNA expression is shown below by analyzing RNA expression via RT-qPCR. Two different vector dilutions were taken along, indicated between brackets. (b) Protein expression of HeLaP4, HeLaP4 LEDGF KD, HeLaP4 LEDGF KD – LEDGF WT/p75 (BC) and HeLaP4 LEDGF KD – LEDGF/p75 E451R-E452R (BC) are shown on top in western blot, while RNA expression is showed below by analyzing RNA expression via RT-qPCR. Different vector dilutions were taken along, indicated between brackets. (c) Protein expression of HeLaP4, HeLaP4 LEDGF KD, HeLaP4 LEDGF KD – LEDGF WT/p75 (BC), HeLaP4 LEDGF KD – LEDGF1-486 (BC) and HeLaP4 LEDGF KD – LEDGF1-486 (BC) are shown on top in western blot, while RNA expression is showed below by analyzing RNA expression via RT-qPCR. Different vector dilutions were taken along, as indicated between brackets.

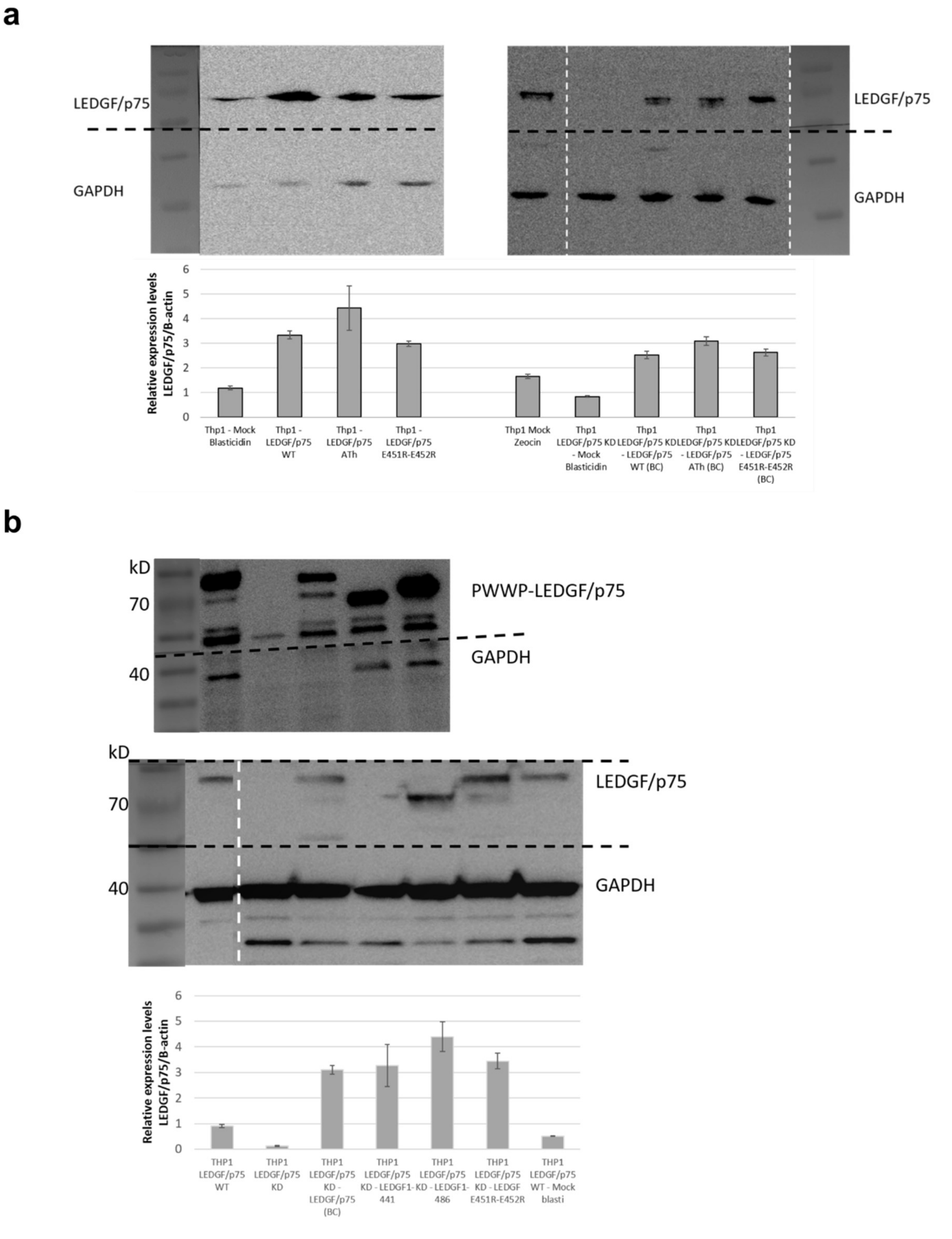

Supporting Figure S5. Expression levels of LEDGF/p75 in Thp1 cells by western blot and qPCR analysis. (a) Protein expression of Thp1 Mock Blasticidin, Thp1 LEDGF/p75 WT overexpression, Thp1 LEDGF/p75 ATh overexpression and Thp1 LEDGF/p75 E451R-E452R overexpression plus back complementation of all these mutants in a THP1 LEDGF KD cell line are shown on top in western blot, while RNA expression is shown below by analyzing RNA expression via RT-qPCR. (b) Protein expression of Thp1 WT cells, Thp1 LEDGF/p75 KD, Thp1 LEDGF/p75 KD – LEDGF/p75 (BC), Thp1

LEDGF/p75 KD – $LEDGF_{1-441}$, Thp1 LEDGF/p75 KD – $LEDGF_{1-486}$ (BC), Thp1 LEDGF/p75 KD – LEDGF/p75 E451R-E452R(BC) and Thp1 Mock Blasticidin are shown on top in western blot. 1 blot is stained with a PWWP-antibody, while the other blot was stained with a LEDGF-C-term antibody. RNA expression of the different mutants is shown below by analyzing RNA expression via RT-qPCR.
